# Sulfur-, nitrogen- and platinum-doped titania thin films with high catalytic efficiency under visible-light illumination

**DOI:** 10.3762/bjnano.9.155

**Published:** 2018-06-04

**Authors:** Boštjan Žener, Lev Matoh, Giorgio Carraro, Bojan Miljević, Romana Cerc Korošec

**Affiliations:** 1 Faculty of Chemistry and Chemical Technology, University of Ljubljana, Večna pot 113, 1000 Ljubljana, Slovenia; 2 Department of Chemical Sciences, University of Padova, Via Francesco Marzolo 1, 35131 Padova, Italy,; 3 University of Novi Sad, Faculty of Technology, Bulevar cara Lazara 1, 21000 Novi Sad, Serbia

**Keywords:** doping, photocatalysis, sol–gel synthesis, thin films, titanium dioxide, visible-light illumination

## Abstract

Titanium dioxide photocatalysts have received a lot of attention during the past decades due to their ability to degrade various organic pollutants to CO_2_ and H_2_O, which makes them suitable for use in environmental related fields such as air and water treatment and self-cleaning surfaces. In this work, titania thin films and powders were prepared by a particulate sol–gel route, using titanium tetrachloride (TiCl_4_) as a precursor. Afterwards, the prepared sols were doped with nitrogen (ammonium nitrate, urea), sulfur (thiourea) and platinum (chloroplatinic acid), coated onto glass substrates by dip-coating, and thermally treated in a muffle furnace to promote crystallization. The resulting thin films were then characterized by various techniques (i.e., TGA-DSC-MS, XRD, BET, XPS, SEM, band gap measurements). The photocatalytic activity of the prepared thin films was determined by measuring the degradation rate of plasmocorinth B (PB), an organic pigment used in the textile industry, which can pose an environmental risk when expelled into wastewater. A kinetic model for adsorption and subsequent degradation was used to fit the experimental data. The results have shown an increase in photocatalytic activity under visible-light illumination of nonmetal and metal doped and co-doped titania thin films compared to an undoped sample.

## Introduction

In recent years, titanium dioxide (TiO_2_) has emerged as one of the most widely investigated semiconductors [1]. Due to its favorable properties (e.g., chemical and biological stability, nontoxicity and inexpensive price) it is used in a variety of applications, such as photovoltaics [2], white pigments [3], electrochemistry [4], catalytic support [5], and most notably, photocatalysis [6]. Photocatalysis is used to describe a process during which a semiconductor (titanium dioxide) interacts with light to produce reactive oxidizing species, which then oxidize adsorbed pollutants, forming CO_2_ and H_2_O as final products [7]. Because of this particularly beneficial characteristic, together with its low-cost and lack of secondary emissions [8], significant research has been focused on the use of TiO_2_ photocatalysis in various environmental applications, such as water treatment [9–11] and air purification [12].

One of the important factors affecting the photocatalytic activity of TiO_2_ is its specific surface area. By increasing the specific surface area (porosity) of TiO_2_, the photocatalytic activity can be increased. One of the ways to increase the porosity of the material is the addition of an organic polymer. After polymer decomposition via thermal treatment, mesoporous materials with high surface area and pore volume are obtained [13–15]. The increase in surface area depends on the type of polymer added, the type of oxides and on the stage of synthesis during which the polymer is added [16–18]. The specific surface area can also be increased by decreasing the particle size of TiO_2_; however, it has been shown that the photocatalytic activity does not monotonically increase with decreasing particle size, but rather there exists an optimal particle size for pure nanocrystalline TiO_2_ [19]. This is due to surface recombination of electron–hole pairs in samples with particle sizes smaller than 6 nm.

One of the main drawbacks of using TiO_2_ as a photocatalyst is the width of its band gap (3.2 eV), which means the electrons can be excited into the conduction band when illuminated by UV light with wavelengths lower than 380 nm [20–22]. Since the solar-light spectrum contains only around 4% of UV light, TiO_2_ is not efficient under solar-light illumination. Different studies have been performed to increase its photocatalytic activity under a broader solar spectrum: doping with metals [23–26], nonmetals [27–30] and co-doping [31–32]. When doping with metals, they act as a free electron trap and thereby prevent electron–hole recombination, which results in enhanced photocatalytic activity of the material system [33–34].

Nitrogen has been shown to be an effective doping element in increasing the response of TiO_2_ during visible-light illumination. When the material is doped with nitrogen, a linear combination of 2p orbitals of N and O results in the formation of hybrid orbitals, causing a narrowing of the band gap by introducing a new energy level close to the valence band. Nitrogen doping can also create impurity energy levels above the valence band, thus effectively also narrowing the band gap [35–36]. Illumination with visible light can then excite electrons from impurity energy levels into the conduction band.

Sulfur doping has also been shown to enhance the photocatalytic activity under visible-light illumination. Sulfur can be doped as an anion (S^2−^), replacing oxygen, or as a cation (S^6+^), thus replacing titanium [37–38]. Substitutional doping of sulfur in TiO_2_ narrows the band gap by the linear combination of the 3p orbitals of S and the 2p orbitals of O [39–40]. In this case, the crystal lattice of TiO_2_ becomes distorted due to the larger ionic radius of S^2−^ compared to O^2−^.

Among all of the approaches to preparation of titania thin films, the sol–gel method is the most versatile, due to its simplicity, cost-effectiveness and low-temperature synthesis. The resulting product is a material with high homogeneity and controllable morphology [8,41–42]. During the sol–gel process, initial hydrolysis of the precursor (e.g., titanium tetrachloride, titanium alkoxides) is followed by polycondensation of the hydroxide monomers, during which –O– and –OH– bridges are established between metallic atoms, resulting in the formation of an oxide and hydroxide network [43].

The aim of this study was to prepare nonmetal and metal-doped as well as co-doped titania thin films, which are photocatalytically active under visible-light illumination. The photocatalytic activity of our samples was determined by measuring the degradation rate of plasmocorinth B (PB), an organic pigment belonging to the group of azo dyes. Azo dyes are used in the textile industry as textile colorants and are stable under normal conditions. They have been shown to damage ecosystems when expelled into water systems, and pose very serious health risks to humans, because they can form aromatic amines, which are potentially carcinogenic substances. Furthermore, when the excess dye is expelled into wastewater after the coloring processes, the water becomes colored, thus decreasing light penetration and consequently decreasing photosynthetic activity of organisms in wastewater [44].

The sols prepared in this work were deposited onto object glass substrates by dip-coating. The immobilization of TiO_2_ in the form of a thin film significantly reduces some of the drawbacks of the practical application of heterogeneous photocatalysis; for instance, the need to separate the photocatalyst from the suspension after the photocatalytic reaction, or the tendency of the particles to agglomerate in aqueous dispersions [45]. The prepared samples were analyzed using thermogravimetric analysis–differential scanning calorimetry–mass spectrometry (TGA-DSC-MS), X-ray diffraction spectroscopy (XRD), specific surface area measurements via Brunauer–Emmett–Teller method (BET), X-ray photoelectron spectroscopy (XPS), scanning electron microscopy (SEM), UV–vis spectroscopy and band gap measurements.

## Results and Discussion

Titania thin films were prepared by a particulate sol–gel synthesis, using titanium tetrachloride (TiCl_4_) as a precursor. During the synthesis, different quantities hydrochloric or sulfuric acid were added. Afterwards, sources of metal and nonmetal dopants and hydroxypropyl cellulose were added. Table 1 shows sample labels, types and amounts of added dopants, thermal treatment temperatures and calculated crystallite sizes for different samples. The measured atomic percentages of dopants were determined by XPS measurements. The platinum in sample S3_N0.5+0.015 M Pt was added as the final layer in the form of chloroplatinic acid by dip-coating. The last number describes molar concentrations of the deposited solution.

**Table 1:** Sample labels, types and amounts of added dopants, thermal treatment temperatures and calculated crystallite sizes for different samples.

Sample name	Dopant; dopant source; amount of added dopant	Nominal amount of dopant relative to TiO_2_ (atom %)	Measured amount (atom %) and type of dopant	Amount (mL) and type of acid added	Thermal treatment temperature (°C)	Calculated crystallite size (nm)

REF	–	–	–	18 HCl	450	39
Urea_5	N; urea; 15.699 mg	5	–	18 HCl	450	39
Urea_10	N; urea; 32.658 mg	10	–	18 HCl	450	50
Urea_15	N; urea; 66.060 mg	15	N; 1	18 HCl	450	41
Urea_20	N; urea; 112.605 mg	20	–	18 HCl	450	50
Thiourea_5	S; thiourea; 19.668 mg	5	–	18 HCl	450	43
Thiourea_10	S; thiourea; 41.329 mg	10	–	18 HCl	450	50
Thiourea_15	S; thiourea; 83.721 mg	15	S; 1	18 HCl	450	60
Thiourea_20	S; thiourea; 142.37 mg	20	–	18 HCl	450	46
S1	S; H_2_SO_4_; 1.65 mL (*c* = 12 M)	–	–	1.65 H_2_SO_4_	600	18
S2	S; H_2_SO_4_; 3.3 mL (*c* = 12 M)	–	–	3.3 H_2_SO_4_	600	10
S3	S; H_2_SO_4_; 4.95 mL (*c* = 12 M)	–	1	4.95 H_2_SO_4_	600	10
S4	S; H_2_SO_4_; 6.6 mL (*c* = 12 M)	–	–	6.6 H_2_SO_4_	600	11
S5	S; H_2_SO_4_; 8.25 mL (*c* = 12 M)	–	–	8.25 H_2_SO_4_	600	12
S3_N0.5	S; H_2_SO_4_; 4.95 mL (*c* = 12 M); N; NH_4_NO_3_; 13.765 mg	– 0.5	S; 1 N; 1	3.3 H_2_SO_4_	600	8
S3_N1	S; H_2_SO_4_; 4.95 mL (*c* = 12 M); N; NH_4_NO_3_; 27.679 mg	– 1	– –	3.3 H_2_SO_4_	600	10
S3_N2	S; H_2_SO_4_; 4.95 mL (*c* = 12 M); N; NH_4_NO_3_; 54.816 mg	– 2	– –	3.3 H_2_SO_4_	600	8
S3_N4	S; H_2_SO_4_; 4.95 mL (*c* = 12 M); N; NH_4_NO_3_; 109.658 mg	– 4	– –	3.3 H_2_SO_4_	600	8
S3_N6	S; H_2_SO_4_; 4.95 mL (*c* = 12 M); N; NH_4_NO_3_; 164.542 mg	– 6	– –	3.3 H_2_SO_4_	600	9
S3_N0.5+1% Pt	S; H_2_SO_4_; 4.95 mL (*c* = 12 M); N; NH_4_NO_3_; 13.765 mg; Pt; H_2_PtCl_6_; 290 μL (*c* =0.344 M)	– 0.5 1	S; 1 N; 1 Pt; 0.5	3.3 H_2_SO_4_	600	8
S3_N0.5+0.015 M Pt	S; H_2_SO_4_; 4.95 mL (*c* = 12 M); N; NH_4_NO_3_; 13.765 mg; Pt; H_2_PtCl_6_; 0.015 M	– 0.5	– –	3.3 H_2_SO_4_	600	–

### Thermal analysis

**Figure 1:**
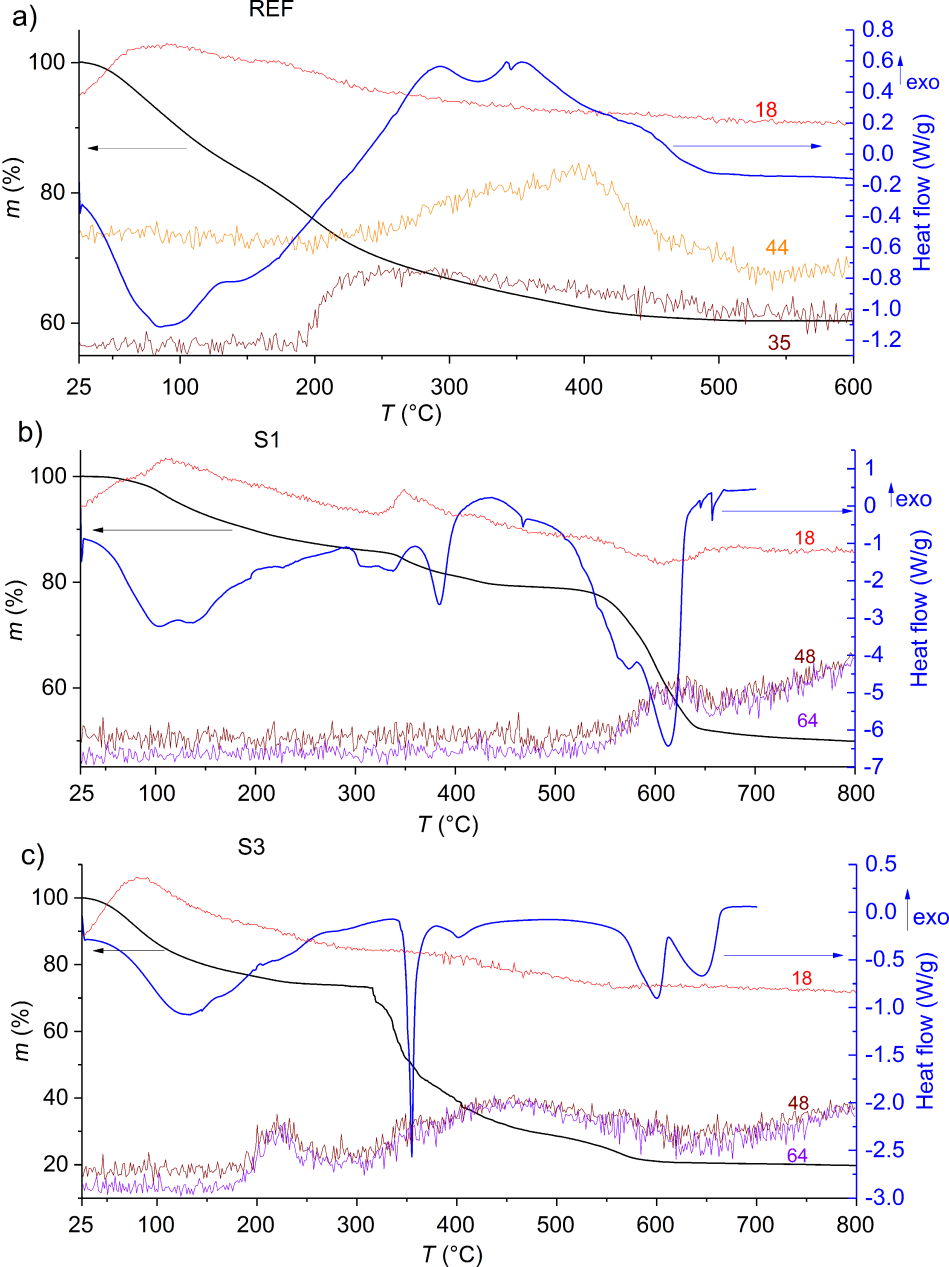
Thermogravimetric analysis–differential scanning calorimetry–mass spectrometry (TGA-DSC-MS) curves of different xerogel samples: a) REF, b) S1 and c) S3.

The purpose of thermal analysis measurements (TGA-DSC-MS) was to determine the crystallization temperature of different xerogel samples and the course of their thermal decomposition. The thermal decomposition of the amorphous xerogels, followed by the complementary techniques of thermal analysis of different samples are shown in Figure 1.

The thermal decomposition of sample REF occurs in three successive, not well-defined steps. In the first step (30–142 °C), water (*m*/*z* = 18) evolves from the sample, whereas during the second (142–296 °C) and third (297–500 °C) steps, chloride ions (most probably in the form of hydrogen chloride (*m*/*z* = 35)) evolve. Furthermore, carbon dioxide (*m*/*z* = 44) also evolved from the sample in the third step. Exothermic peaks in the DSC curve from ≈300 °C onward are due to the process of crystallization of the sample. Around 450 °C, the mass loss and exothermic processes are completed, meaning that the final oxide material is formed. Based on these results and the results of XRD measurements, we determined 450 °C to be the optimal temperature for the thermal treatment of samples synthesized with HCl.

The thermal decomposition of sample S1 also occurs in three successive steps. In the first (30–230 °C) and second (330–440 °C) step, water (*m*/*z* = 18) evolves, whereas in the third (510–560 °C) step, sulfur oxides (*m*/*z* = 48 and 64) evolve. Exothermic peaks, due to to the process of crystallization are overlapped by endothermic peaks, originating from the evolution of sulfates. Based on these results and the results of XRD measurements, 600 °C was determined to be the optimal temperature for the thermal treatment of samples synthesized with H_2_SO_4_.

The thermal decomposition of sample S3 occurs in three successive steps. In the first step (30–250 °C), water (*m*/*z* = 18) evolves from the sample, in the second (285–510 °C) and third (510–610 °C) step sulfur oxides (*m*/*z* = 48 and 64) evolve from the sample. The exothermic peaks of crystallization are again overlapped by the endothermic peaks due to the evolution of sulfates. Sulfur oxides start evolving from this sample at lower temperatures (compared to S1) due to the presence of excess sulfates that are not bound in the structure of TiOSO_4_. We attribute this to higher added volumes of H_2_SO_4_ during the synthesis. In the last step, sulfates from the thermal decomposition of TiOSO_4_ evolve from the sample.

### X-ray diffraction

**Figure 2:**
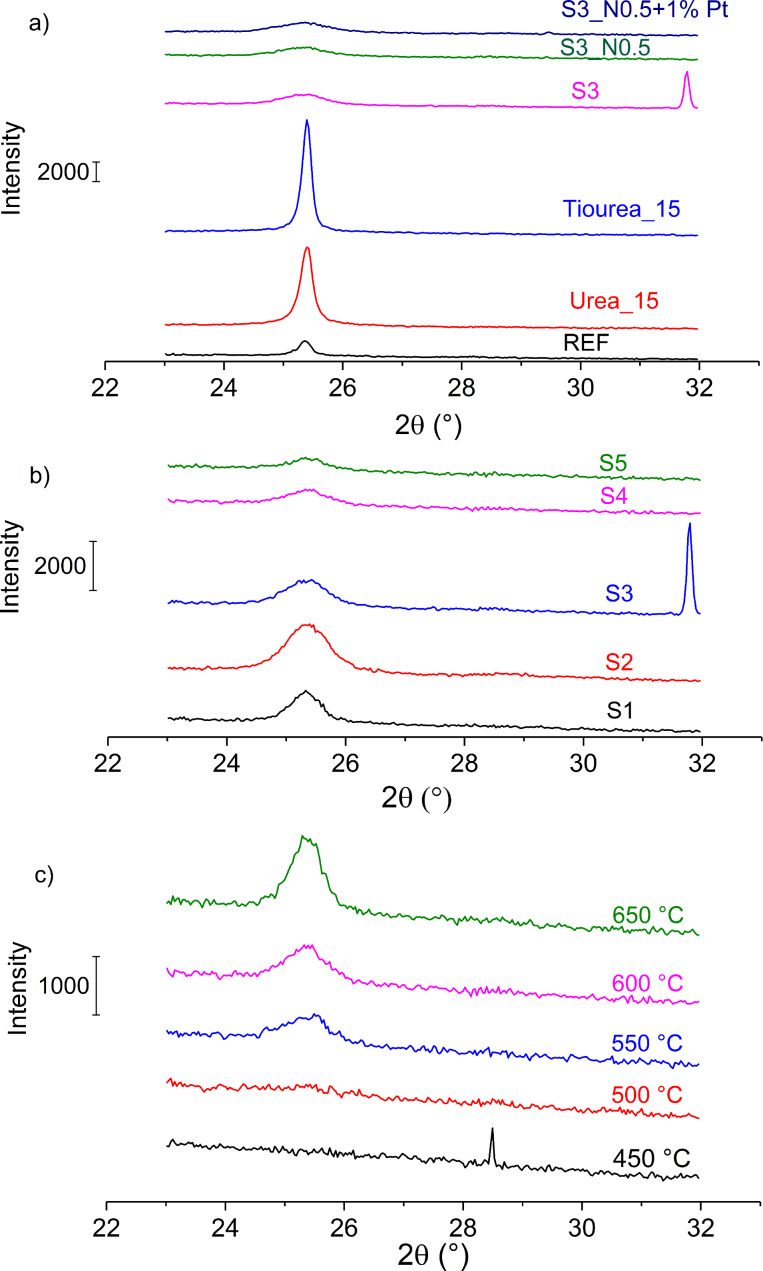
X-ray diffraction patterns for samples a) synthesized with HCl, b) samples S1–S5, and c) the crystallization process of sample S4.

Typical XRD patterns for selected samples, synthesized with HCl (thermal treatment at 450 °C) or H_2_SO_4_ (thermal treatment at 600 °C), S1–S5 and the structural evolution of sample S4 as a function of thermal treatment temperature are shown in Figure 2. All of the samples have one major peak at 2θ ≈ 25.3°, which corresponds to the reflections of anatase phase. Some patterns also show peaks at 2θ ≈ 29° and 2θ ≈ 32°, which correspond to reflections of silicon in the case of very thin films, which do not fully cover the substrate. Anatase is the only polymorphic modification of TiO_2_ present in the samples.

Table 1 shows the average calculated crystallite size of anatase in the thin films, calculated with Scherrer’s formula (Equation 1), for different samples.

[1]
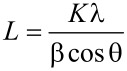


In Equation 1, *L* represents the calculated crystallite size, *K* is a dimensionless shape factor, λ is the X-ray wavelength, β is the peak width at half the maximum intensity (FWHM) and θ is the Bragg angle.

Larger crystallites were found in samples synthesized with HCl (REF–Thiourea_20, 39–60 nm) compared to samples synthesized with H_2_SO_4_ (S1–S5, 10–18 nm). The results also show a trend of decreasing crystallite size with increasing volume of H_2_SO_4_ added during the synthesis (S1–S5). We contribute this to the formation of titanium oxysulfate (TiOSO_4_) which inhibits crystallization. The addition of NH_4_NO_3_ also decreases the crystallite size of the samples. The addition of Pt did not have any effect on the crystallite size.

**Crystallization of sample S4:** Figure 2c shows XRD patterns for sample S4 at different thermal treatment temperatures. At 450 and 500 °C, the sample has not yet crystallized (amorphous phase present). The sample starts crystallizing at 550 °C (crystallite size 9 nm). With increasing thermal treatment temperature, the crystallite size also increases (11 nm at 600 °C and 14 nm at 650 °C).

### Specific surface area (BET)

Table 2 summarizes the BET specific surface area measured for selected samples. The samples synthesized with H_2_SO_4_ (samples S3–S3_N0.5+1% Pt) have a higher specific surface than samples synthesized with HCl (REF–Thiourea_15). We can explain this with the observed decrease in crystallite size, which is typical for samples synthesized with H_2_SO_4_ (≈10 nm) compared to samples synthesized with HCl (≈50 nm) [19].

**Table 2:** BET specific surface area and band gap values for selected samples.

Sample name	Surface area (m^2^/g)	Band gap (eV)	Wavelength (nm)

REF	44.1	3.44	361
Urea_15	24.2	3.16	392
Thiourea_15	49.5	2.81	442
S3	80.2	3.10	400
S3_N0.5	84.5	3.07	403
S3_N0.5+1% Pt	83.7	3.08	403

**Figure 3:**
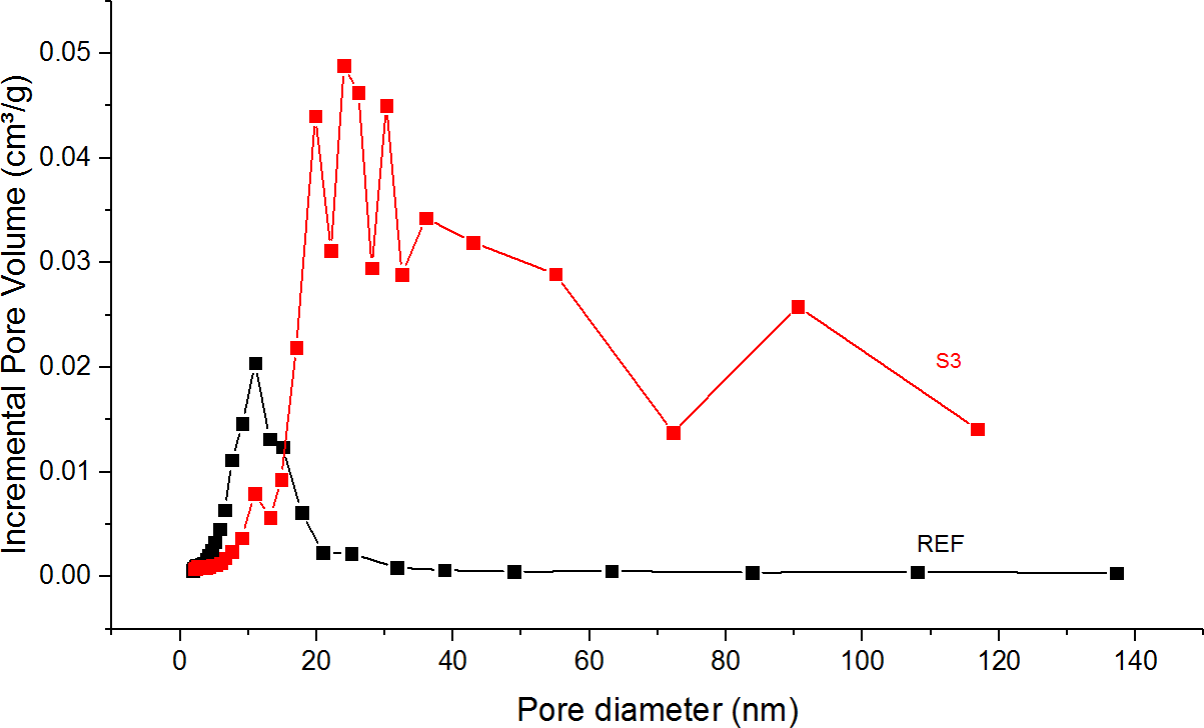
Pore size distribution for samples REF and S3.

Figure 3 shows the pore size distribution for a sample synthesized with HCl (REF) and H_2_SO_4_ (S3). Sample REF has a well-defined uniform pore size distribution (≈10 nm), whereas S3 has a multimodal pore size distribution. The distributions of pores in other samples synthesized with HCl (Urea_15, Thiourea_15) and H_2_SO_4_ (S3_N0.5, S3_N0.5+1% Pt) are similar to the distributions shown in Figure 3.

### Band gap measurements

**Figure 4:**
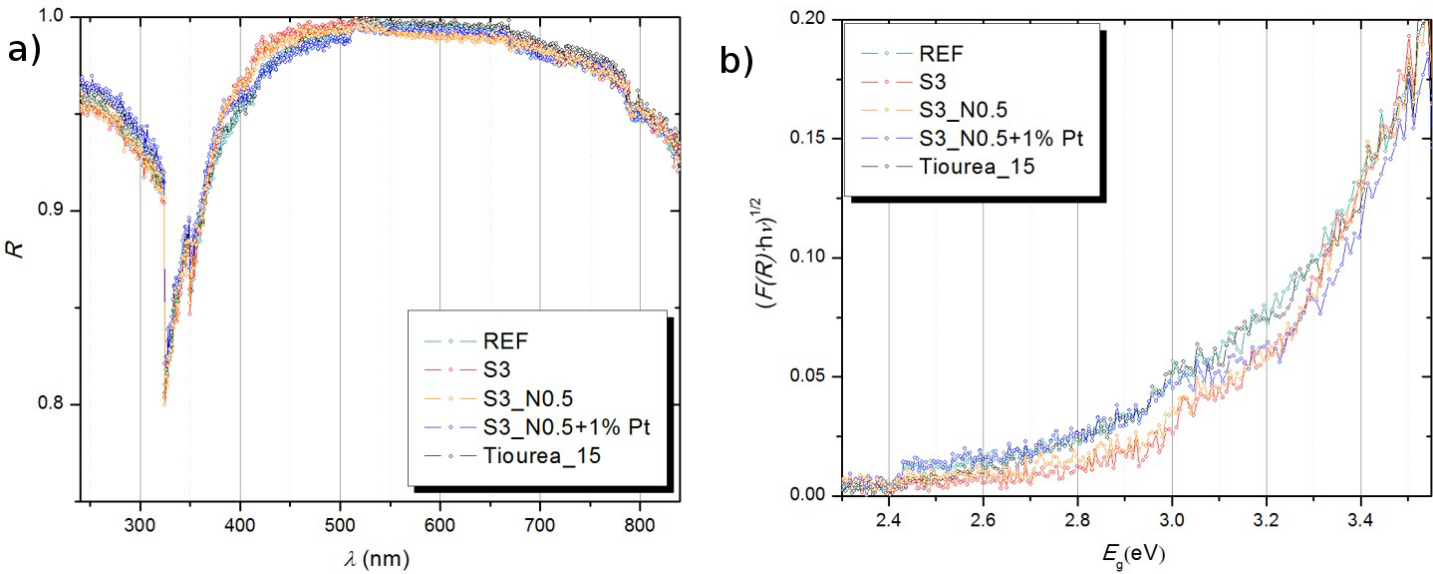
(a) Measured diffuse reflectance plotted as a function of the wavelength – *R*(λ) and (b) modified Kubelka–Munk function vs energy.

The diffuse reflectance, *R*, of the measured samples as a function of the wavelength λ is shown in the Figure 4a, and in Figure 4b, one can see the modified Kubelka–Munk function, (*F*(*R*)*h*ν)^n^, vs energy, *E*_g_, where *F* is the Kubelka–Munk function of the measured diffuse reflectance *R* as follows: *F*(*R*) = (1 − *R*)^2^ / 2*R*. The band gap energy was determined from this plot by extrapolating the linear fit of the straight section to the (*F*(*R*)*h*ν)^n^ = 0 intercept of the energy coordinate.

Table 2 shows band gap values for selected samples. The results show an expected narrowing of the band gap width for doped samples (Urea_15, Thiourea_15, S3, S3_N0.5 and S3_N0.5+1% Pt) relative to the undoped sample (REF). Consequentially, doping TiO_2_ with S or N shifts the absorbance toward visible light, whereas doping with Pt (sample S3_N0.5+1% Pt) does not additionally narrow the band gap compared to the sample without Pt (S3_N0.5). The band gap narrowing is explained by the creation of new extra states within the band gap created by interstitially doped N and S [18]. On the other hand, Pt acts as a trap for free electrons and inhibits electron–hole recombination. Doping our reference sample with urea and thiourea has shown to significantly decrease the band gap width (band gap is 3.44 eV for REF, 3.16 eV for Urea_15 and 2.81 eV for Thiourea_15).

### X-ray photoelectron spectroscopy

**Figure 5:**
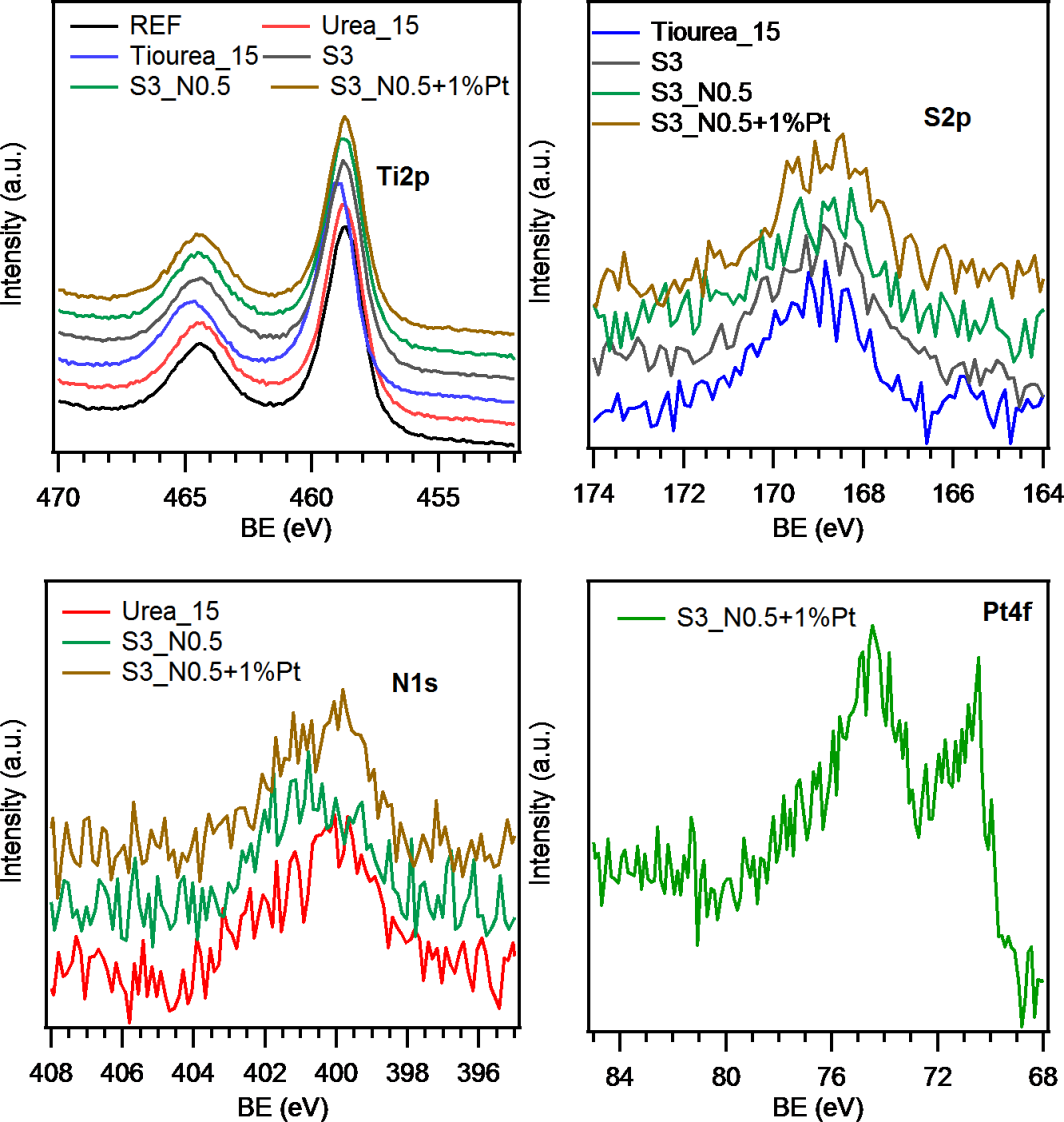
X-ray photoelectron spectra for selected specimens, reporting the Ti 2p, S 2p, N 1s and Pt 4f peaks.

XPS measurements were performed on selected samples to determine the amount of dopant present and the oxidation states. XPS spectra for selected samples are presented in Figure 5. The survey spectra of the specimen (not reported) indicate the presence of carbon, oxygen and nitrogen in all the analyzed samples. Irrespective of the synthesis conditions, the C 1s peak (not reported) is located at 284.8 eV, which can be attributed to adventitious surface contamination due to air exposure [46]. All the specimens are characterized by the Ti 2p_3/2_ binding energy (BE) of 458.7 eV, as well as the separation between the spin–orbit components (Δ(BE) = 5.7 eV) (Figure 5 top-left) in line with the presence of Ti(IV) in TiO_2_ [46]. Regarding the sulfur containing samples (Figure 5, top right panel), the S 2p XPS peaks located at 168.7 eV with a tailing at higher binding energies could be attributed to S(VI) species corresponding to the S(VI) cation in S=O and S–O bonds, occurring when Ti(IV) is interstitially replaced by sulfur atoms. In addition, no signal for anionic S species are recorded [46–47]. Irrespective of the synthetic conditions, the analysis of the N 1s peaks (Figure 5 bottom, left panel) highlights the presence of a component located at BE = 400.1 eV, suggesting the formation of interstitially oxidized nitrogen in Ti–O–N species. The N 1s signal is characterized by a high signal-to-noise ratio, preventing an unambiguous attribution of the peak tailing at higher/lower energies, which are attributed to the absorption of other nitrogen species [46]. On the other hand, the presence of Ti–N bonds can be clearly ruled out [46,48]. In the sample S3_N0.5+1%Pt, the Pt 4f_7/2_ photoelectron peak is centered at BE = 71.0 eV, confirming the presence of elemental Pt [46]. Typical surface concentration values calculated by XPS analyses are 1 atom % for S and N, whereas platinum has a concentration of 0.5 atom %.

### Scanning electron microscopy

**Figure 6:**
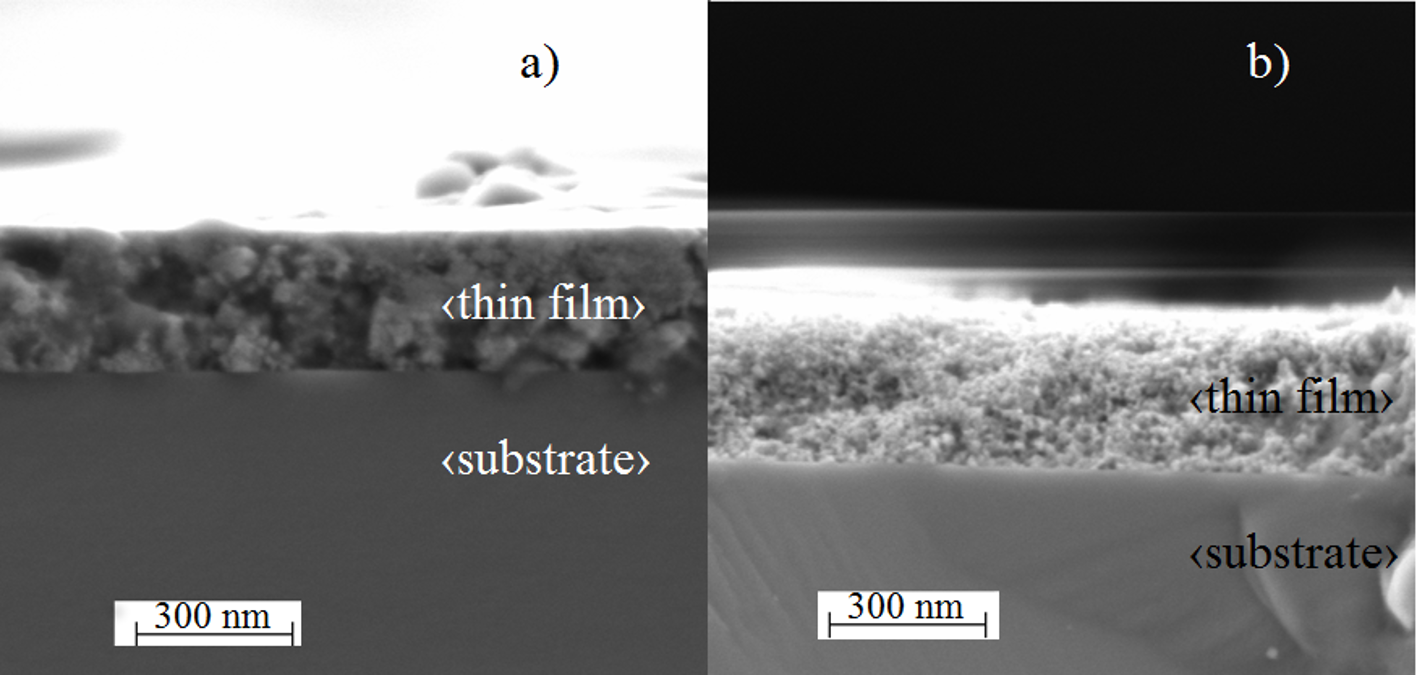
Cross-sections of thin films of samples a) Urea_15 and b) S4.

Figure 6 shows cross-sections of thin films of Urea_15 (Figure 6a) and S4 (Figure 6b). By substituting HCl (Figure 6a) with H_2_SO_4_ (Figure 6b), we obtained more porous (confirmed by BET measurements) and thicker (thickness is 210 nm for Urea_15 and 280 nm for S4) thin films. Figure 5a also shows the formation of TiO_2_ agglomerates approximately 50 nm in size, which has also been observed with XRD.

### Photocatalytic activity

Due to the large number of samples prepared, only the results related to samples with optimal photocatalytic activity will be presented here. The photocatalytic activity under visible light was measured by observing the degradation rate of PB, an organic dye. The concentration of PB at different illumination times was determined by measuring its UV–vis absorbance spectra and calculating using the Beer–Lambert law (Equation 2).

[2]
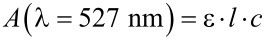


In this equation, *A* is the absorbance of the PB solution at 527 nm excitation at different illumination times, *c* is the molar concentration of PB at different illumination times, ε is the molar absorptivity coefficient and *l* is the optical path length.

**Figure 7:**
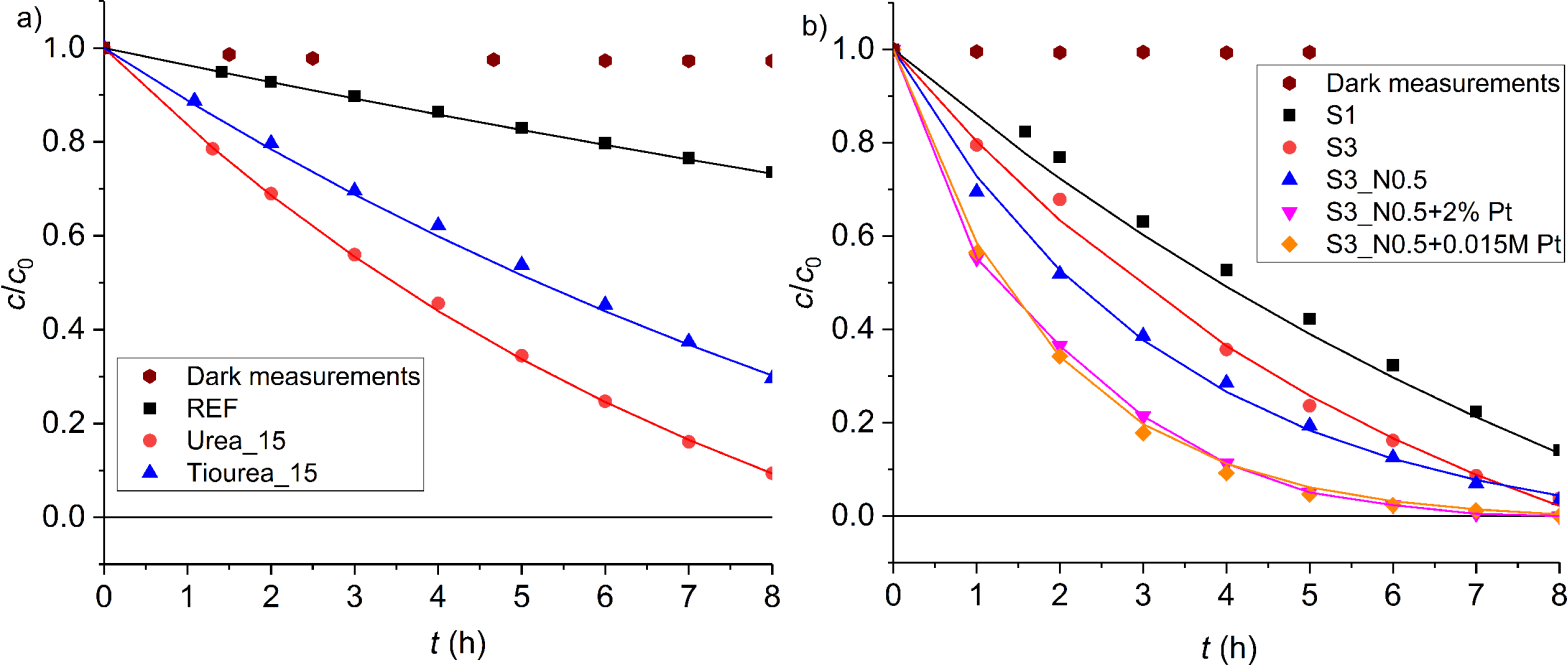
Photocatalytic activity of different samples a) synthesized with HCl; dark measurements performed for sample REF and b) synthesized with H_2_SO_4_; dark measurements performed for sample S3.

The results are presented in Figure 7, which show the kinetic results for plasmocorinth B (PB) degradation for samples synthesized with HCl (Figure 7a) and synthesized with H_2_SO_4_ (Figure 7b). Different dots represent experimental data (acquired with UV–vis spectroscopy) and lines represent a fitted kinetic model. A kinetic model for adsorption (zero-order rate constant, *k*_0_) and subsequent degradation (first-order rate constant, *k*_1_) was used to fit the experimental data as follows:

[3]
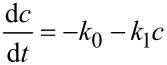


By solving the differential Equation 3, we get the following model equation describing the dependence of concentration on time:

[4]
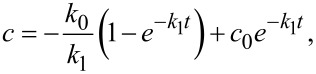


where *c* represents the concentration of PB after a certain time of illumination, *c*_0_ represents the concentration of PB before illumination and *t* is the illumination time.

To separate the processes of adsorption and photocatalysis, two experiments were carried out under dark conditions. The results of these experiments are presented only as data points (filled brown circles) in Figure 7a (Adsorption REF) and Figure 7b (Adsorption S3). We observed only a small decrease in the concentration of PB due to adsorption.

**Figure 8:**
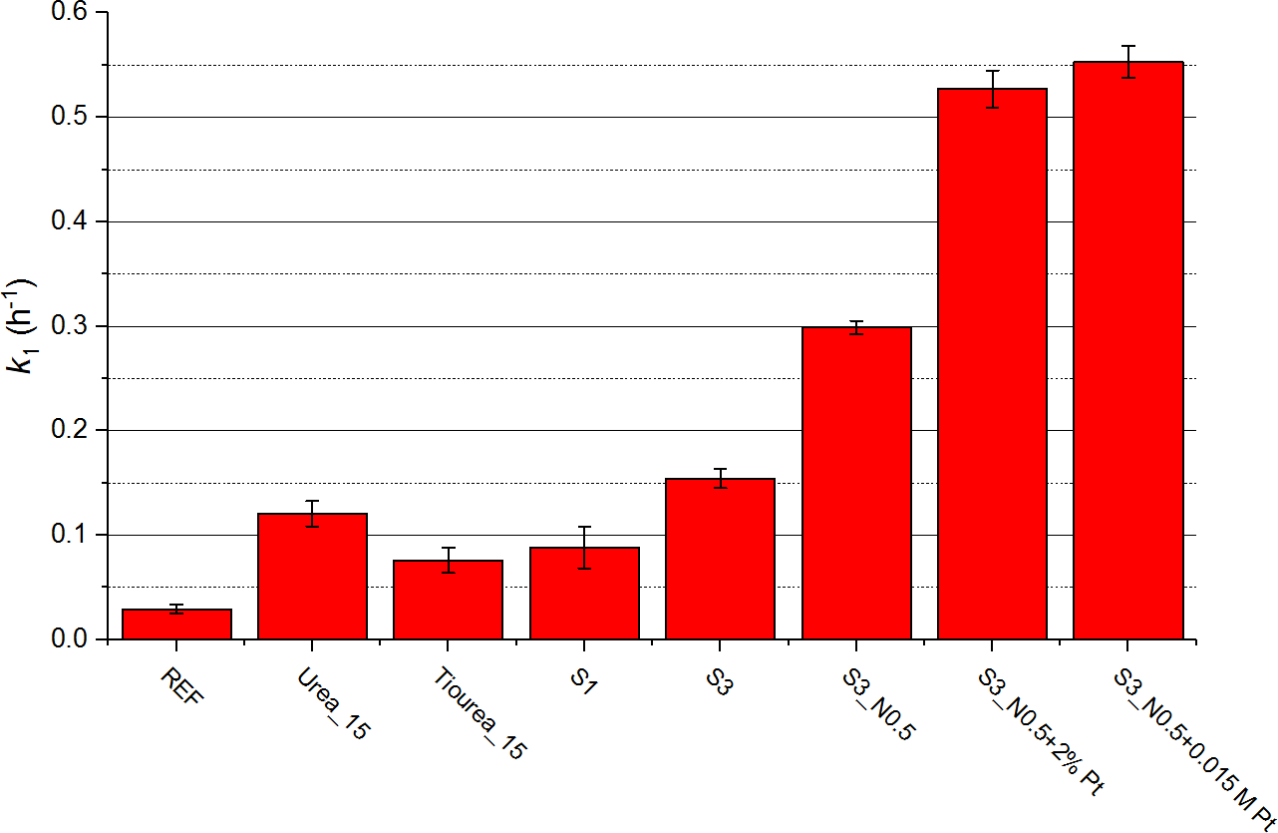
First-order rate constant for plasmocorinth B degradation for selected photocatalysts synthesized in this work.

Figure 8 shows the first-order rate constants (*k*_1_) for PB degradation calculated using the model presented in Equation 4, whereby higher values correspond to higher photocatalytic activity of the sample.

By doping the reference sample (REF) with N (sample Urea_15) and S (sample Thiourea_15) (confirmed by XPS measurements), we were able to significantly decrease the band gap width of our material (3.44 eV for REF, 3.16 eV for Urea_15 and 2.81 eV for Thiourea_15) and consequently increase the degradation rate of PB (*k*_1_ = 0.029 h^−1^ for REF, 0.12 h^−1^ for Urea_15 and 0.076 h^−1^ for Thiourea_15). However, the photocatalytic activity of these doped samples is limited by their relatively small specific surface area (24.2 m^2^/g for Urea_15 and 49.5 m^2^/g for Thiourea_15).

By substituting HCl with H_2_SO_4_ we were able to prepare more porous samples (surface area for S3 equals 80.2 m^2^/g) with a narrower band gap relative to REF (band gap for S3 is 3.10 eV). We contribute the band gap narrowing to H_2_SO_4_ acting as a source of doped S (confirmed by XPS measurements). Furthermore, both acids also accelerate the dissolution of the precursor during sol–gel synthesis and act as sol stabilizers. Consequently, the PB degradation rate for S3 is higher than any other sample synthesized with HCl (*k*_ 1_ = 0.15 h^−1^ for S3).

Samples co-doped with N and S (e.g., S3_N0.5) have shown an increased photocatalytic activity under visible-light illumination (*k*_1_ for S3_N0.5 is 0.30 h^−1^). The band gap width for this sample was shown to be narrowed (3.07 eV). Researchers attribute this to a synergetic effect between N 2p and S 3p orbitals, resulting in a hybridization of the two orbitals generated close to the valence band [31,49]. Furthermore, with the addition of NH_4_NO_3_, the sample became more porous (surface area of 84.5 m^2^/g), which also explains the increased photocatalytic activity.

The band gap width and porosity have not changed significantly with the addition of Pt (band gap of 3.08 eV; surface area of 83.7 m^2^/g). However, the PB degradation rate for these samples increased significantly, which can be explained by Pt acting as an efficient free electron trap, thereby reducing the undesirable electron–hole recombination while also improving the free-electron transfer to the adsorbed PB [25,50]. The best photocatalytic activity was observed for samples S3_N0.5+2% Pt (*k*_1_ = 0.53 h^−1^) and S3_N0.5+0.015M Pt (*k*_1_ = 0.55 h^−1^).

## Conclusion

Nitrogen (N), sulfur (S) and platinum (Pt)-doped titania thin films were prepared by a particulate sol–gel synthesis. An organic polymer (hydroxypropyl cellulose; HPC) was added to increase the porosity of the samples. The prepared samples were then deposited onto glass substrates by dip-coating and thermally treated at 450 and 600 °C. By substituting HCl with H_2_SO_4_ during the sol–gel synthesis, we were able to obtain samples doped with S, which resulted in samples with a higher porosity, smaller crystallite size and a narrower band gap. All of this resulted in an increase in photocatalytic activity. Additionally, doping these samples with N slightly increased the surface area and narrowed the band gap, which consequentially increased its photocatalytic activity. The addition of Pt did not significantly affect the surface area or band gap width, but nevertheless increased the photocatalytic activity. We contribute this to Pt acting as a free-electron trap, thus reducing the recombination rate. To summarize, by doping our reference sample with N, S or Pt we were able to significantly increase the photocatalytic activity of titania thin films under visible-light illumination.

## Experimental

The chemicals in this study were used as purchased: titanium tetrachloride (TiCl_4,_ >98%) from Fluka; hydrochloric acid (HCl, 37%) from Fluka; sulfuric acid (H_2_SO_4_, 97%) from Sigma-Aldrich; ethanol absolute; ammonium nitrate (NH_4_NO_3_) from Zorka Šabac; hydroxypropyl cellulose (HPC, *M*_w_ = 100.000 g/mol) from Sigma-Aldrich; plasmocorinth B (PB, dye content ≈60%) from Sigma-Aldrich; thiourea (pro analysis) from Kemika and urea (98%) from Acros Organics.

### Synthesis

Titanium dioxide was prepared by a particulate sol–gel synthesis from titanium tetrachloride (TiCl_4_) precursor. In the first step of the synthesis, the precursor was dissolved in water, which was previously acidified with different volumes of concentrated hydrochloric (HCl) and 12 M solution of sulfuric acid (H_2_SO_4_). After the dissolution, different chemicals, which acted as sources of metal and nonmetal dopants (e.g., urea, thiourea, ammonium nitrate – NH_4_NO_3_, chloroplatinic acid – H_2_PtCl_6_) and hydroxypropyl cellulose (HPC, an organic polymer, which increases the porosity of materials) were added. After deposition on glass substrates with dip-coating, the thin films were thermally treated in a muffle furnace at 450 (for the synthesis involving HCl) and 600 °C (for the synthesis involving H_2_SO_4_) for 30 minutes to promote crystallization of TiO_2_.

**Synthesis involving HCl (doping with urea and thiourea):** 5.52 mL of TiCl_4_ was dissolved in a solution, containing 90 mL of distilled water and 18 mL of concentrated HCl. After a day of stirring, 0.3 wt % (relative to the mass of the sol) of HPC was added. These samples were then doped with urea and thiourea. The undoped sample was named REF, whereas the doped samples were named Urea_5; Urea_10; Urea_15 and Urea_20. The numbers describe the molar percentage of the added dopant (relative to TiO_2_). The same process was performed for thiourea.

**Synthesis involving H_2_SO_4_ (doping with NH_4_NO_3_ and H_2_PtCl_6_):** 3.68 mL of TiCl_4_ were dissolved in a solution containing 60 mL of distilled water and different volumes of 12 M H_2_SO_4_. After one day of stirring, 0.3 wt % (relative to the mass of the solution) of HPC was added. Table 3 shows the sample names and corresponding volume of 12 M H_2_SO_4_ added.

**Table 3:** Sample names and corresponding volume of 12 M H_2_SO_4_ added.

Sample name	Added H_2_SO_4_ (mL)

S1	1.65
S2	3.30
S3	4.95
S4	6.60
S5	8.25

Sample S3 was then doped with different amounts of NH_4_NO_3_. The samples were named S3_N0.25, S3_N0.5, S3_N1, S3_N2, S3_N4 and S3_N6. The numbers next to N describe the molar percentages of added NH_4_NO_3_ (relative to TiO_2_).

Finally, we added H_2_PtCl_6_ to sample S3_N0.5, in order to dope it with platinum. Some of the samples had platinum added directly into the sol (S3_N0.5+0.5% Pt, S3_N0.5+1% Pt and S3_N0.5+2% Pt), where the percentage describes the molar percentage of added Pt (also relative to TiO_2_). To other samples (S3_N0.5+0.0025M Pt to S3_N0.5+0.02M Pt), platinum was added as the final layer in the form of chloroplatinic acid by dip-coating. The numbers refer to the molar concentration of deposited solution of H_2_PtCl_6_.

For XRD and XPS characterization, thin films were deposited onto silicon resins and thermally treated at 450 (for the synthesis involving HCl) and 600 °C (for the synthesis involving H_2_SO_4_) for 30 minutes.

Powder analogues for BET and band gap analysis were prepared by drying the sols in air to produce xerogels. The xerogels were then thermally treated in a muffle furnace under the same conditions as the thin films.

### Characterization

Simultaneous TGA–DSC–MS measurements were performed on a Mettler Toledo TGA/DSC1 instrument, coupled to a Pfeiffer Vacuum ThermoStar mass spectrometer. The samples were placed into Pt crucibles and heated from 25 to 800 °C at a heating rate of 10 °C/min.

XRD patterns were measured with a Siemens D5000 instrument in the 2θ range of 23–32°. The average crystallite sizes were calculated using Scherrer’s equation.

The BET specific surface area and pore size distribution of the powders were determined by measuring the nitrogen adsorption–desorption isotherms with a Tristar 3000, Micromeritics (USA) instrument. The measurements were performed at −196 °C (77 K). The samples were outgassed under vacuum for 16 h at 110 °C (383 K). The mass of the samples in the analyzer was ≈0.1 g. The specific surface area was calculated from the adsorption measurements in the relative pressure (*p*/*p*_0_) range of 0.05–0.25.

XPS spectra were recorded on a Perkin Elmer φ 5600ci spectrometer at a pressure lower than 10^−8^ mbar, using a non-monochromatized Al Kα excitation source (*h*ν = 1486.6 eV). The BE shifts were corrected by assigning a value of 284.8 eV to the C 1s line of adventitious carbon [51].

UV–vis absorption spectra were collected using an Agilent Cary 60 UV–vis spectrophotometer in the wavelength range λ = 400–800 nm.

The morphology of thin films was examined using a field emission scanning electron microscope (FEI Inspect™ F50 and Ultra Plus Zeiss).

The band gap measurements were performed by measuring the reflectance of the samples using a UV–vis spectrophotometer (Evolution 600, Thermo Scientific), using the diffuse reflectance accessory (DRA-EV-600) integrating sphere. The measurements were performed in the range 240–840 nm a step of 1 nm and speed of 10 nm min^−1^. The material Spectralon^®^ was used as a reference. The band gap energy of the measured samples was determined using the Tauc’s plot [52], based on the Kubelka–Munk theory [53–54].

### Photocatalytic activity tests

The photocatalytic efficiency of the thin films was determined by measuring the degradation rate of plasmocorinth B (PB, γ = 12 mg/L). The photocatalytic activity of the prepared thin films was tested in a home-made photoreactor, fitted with six 15 W Lumilux de Luxe daylight lamps. The thin films with an average area of 96 cm^2^ were immersed into a batch reactor, which was filled with 70 mL of dye solution and was purged with oxygen (flow rate 100 mL/min). The activity of the thin films was determined by measuring the absorption spectra of PB (absorption maximum at ≈527 nm) after different durations of illumination with visible light. The concentration of the dye was calculated using the Beer–Lambert law.
